# A meta-analysis of randomized controlled trials comparing the efficacy and safety of anastrozole *versus* tamoxifen for breast cancer

**DOI:** 10.18632/oncotarget.16466

**Published:** 2017-03-22

**Authors:** Yan Yang, Wei Pan, Xinyu Tang, Shuqing Wu, Xinchen Sun

**Affiliations:** ^1^ Department of Radiation Oncology, The First Affiliated Hospital of Nanjing Medical University, Nanjing, China; ^2^ Department of Radiation Oncology, The Affiliated Jiangning Hospital of Nanjing Medical University, Nanjing, China; ^3^ Department of Radiation Oncology, The Affiliated Danyang Hospital of Nantong University, Nantong, China

**Keywords:** breast cancer, anastrozole, tamoxifen, meta-analysis

## Abstract

Whether anastrozole has superior effects to tamoxifen for breast cancer remains controversial. Therefore, we conducted this meta-analysis of randomized controlled trials (RCTs) to compare the efficacy and safety of anastrozole *versus* tamoxifen as adjuvant therapy in breast cancer. A systematic literature search of PubMed, Web of Science, Embase, and Cochrane library were performed to evaluate the survival benefits and toxicity profiles of patients with breast cancer who were treated with anastrozole or tamoxifen. The main outcome measures included disease-free survival (DFS), recurrence-free survival (RFS), overall survival (OS), overall response rate (ORR), and adverse events. Hazard ratios (HRs) or risk ratios (RRs) with 95% confidence intervals (CIs) were pooled using a fixed-effects model or random-effects model. Nine RCTs with a total of 15,300 patients met the inclusion criteria and were included in this meta-analysis. Pooled estimates suggested that, anastrozole was associated with a significantly improvement in DFS (HR=0.72, 95%CI: 0.55-0.94; *P*=0.016), and ORR (RR=1.21, 95% CI: 1.05-1.39; *P*=0.009) than tamoxifen. But it did not prolong OS (HR=0.96, 95%CI: 0.77-1.21; *P*=0.751). Compared with tamoxifen, anastrozole induced a higher incidence of arthralgia (RR=1.55, 95%CI: 1.20-1.99; *P*=0.001) and bone pain (RR=1.31, 95%CI: 1.05-1.62; *P*=0.015), as well as a lower incidence of vaginal bleeding (RR=0.51, 95%CI: 0.28-0.93; *P*=0.029), vaginal discharge (RR=0.31, 95%CI: 0.12-0.82; *P*=0.017), and thromboembolic events (RR=0.39, 95%CI: 0.28-0.55; *P*<0.001). Based on the current evidence, patients with breast cancer would benefit from the anastrozole treatment.

## INTRODUCTION

Breast cancer is the most common cancer in women worldwide, with an estimated 1.6 million new cases every year [1]. The treatment options for breast cancer vary depending on histological grade, tumor characteristics, and extent of disease [2]. For premenopausal women with oestrogen receptor (ER)-positive or progesterone receptor (PgR)-positive breast cancer, the treatment strategies include ablative surgery [3], radiotherapy [4, 5], cytotoxic chemotherapy, or adjuvant endocrine [6]. The endocrine treatment includes ER antagonist tamoxifen, and luteinisting hormone releasing hormone (LHRH) agonists such as goserelin [7].

Tamoxifen is a selective ER modulator, and it largely or wholly binds to the receptor protein [8]. Previous studies suggest that, treatment with 5 years of tamoxifen could reduce local, contralateral, and distant recurrence rates, as well as decrease the 15-year breast mortality in 75% to 80% of patients who had ER positive breast cancer [9, 10]. In the National Surgical Adjuvant Breast and Bowel Project (NSABP) B-24 trial [6], all patients with ductal carcinoma *in situ* (DCIS) were randomly allocated to receive tamoxifen or matching placebo. At the median follow up of 6 years, tamoxifen significantly reduced the recurrence rate by 37% as compared with placebo [6]. Retrospective evaluation of the 732 patients demonstrated that tamoxifen was associated with a 51% reduction in subsequent breast cancer for women with ER-positive DCIS, but no effect in ER-negative patients [11]. Furthermore, in the UK/ANZ DCIS trial [12], 1578 women with locally excised DCIS received the treatment of tamoxifen with or without radiotherapy. After a median follow up of 12.7 years, the rate of new breast cancer events was significantly reduced by 29% [12]. Tamoxifen also had effects on the reduction of ipsilateral DCIS recurrence, but not the ipsilteral invasive recurrence [12].

Recently, the third-generation aromatase inhibitors have shown beneficial effects in the management of women with early stage breast cancer. And in 2004, the American Society of Clinical Oncology (ASCO) Technology Assessment recommended that, an aromatase inhibitor should be included to reduce the risk of tumor recurrence when treating the hormone-sensitive early-stage breast cancer [13].

Whether the third-generation aromatase inhibitor, anastrozole, has superior effects to tamoxifen in breast cancer remains controversial. To increase power and precision, we conducted this meta-analysis based on relevant randomized controlled trials (RCTs) to compare the efficacy and safety of anastrozole *versus* tamoxifen as adjuvant therapy in the treatment of women with breast cancer.

## MATERIALS AND METHODS

### Literature search

We conducted a comprehensive search to identify RCTs that compared anastrozole *versus* tamoxifen in women with breast cancer. Four Databases, including PubMed, Embase, Web of Science, and Cochrane library, were systematic reviewed from inception to November 25, 2016. Search terms used were listed as the followings: (“breast neoplasms” [MeSH Terms] OR (“breast” [All Fields] AND “neoplasms” [All Fields]) OR “breast neoplasms” [All Fields] OR (“breast” [All Fields] AND “cancer” [All Fields]) OR “breast cancer” [All Fields]) AND (“anastrozole” [Supplementary Concept] OR “anastrozole” [All Fields]) AND (“tamoxifen” [MeSH Terms] OR “tamoxifen” [All Fields]). The search was limited to human subjects and RCTs, and no language restriction was imposed. We also searched the ClinicalTrials.gov registry and manually checked the reference lists of the previous reviews and selected articles to identify other potential articles.

### Study inclusion

Published RCTs that met the following criteria were included: (1) study design: RCT; (2) population: women with breast cancer; (3) intervention: anastrozole; (4) comparison: tamoxifen; (5) outcomes: disease-free survival (DFS), recurrence-free survival (RFS), overall survival (OS), overall response rate (ORR), adverse events. When the same population was appeared in several publications, we only included the one with latest or most comprehensive information.

### Data extraction

Two investigators (Yan Yang and Wei Pan) independently extracted the following information from the included studies: first author's name, year of publication, sample size, patients’ demographic characteristics, hormone-receptor status, duration of follow up, hazard ratio (HR) with 95% confidence intervals (95%CIs) for DFS, RFS, OS, and incidence of adverse events. Disagreements between the investigators were resolved by discussion and consensus.

### Risk of bias assessment and grading quality of evidence

Two investigators (Yan Yang and Xinyu Tang) independently assessed the risk of bias in included studies, using the method recommended by Cochrane Collaboration [14]. We considered each trial as high, low, or unclear risk of bias according to the following criteria: random sequence generation; allocation concealment; blinding of outcome participants and personnel; blinding of outcome assessment; incomplete outcome data; selective reporting and other bias.

The quality of evidence for the outcome measures was evaluated using the Grading of Recommendations Assessment, Development and Evaluation (GRADE) approach [15]. A summary table was constructed using the GRADE Profiler (version 3.6, GRADE Epro).

### Statistical analysis

We calculated HR with 95%CIs for time-to-event variables, and risk ratios (RRs) with 95%CIs for dichotomous outcomes. Before the data were synthesized, we first tested the heterogeneity between the included studies using *I*^2^ statistic and Cochrane Q chi-square test [16]. The studies were considered to have significant statistical heterogeneity when the value of *I*^2^ was more than 50%, or the value of P was less than 0.10 [16]. When heterogeneity was found among the included studies, a random-effects model (DerSimonian-Laird method) [17] was used to pool the estimates; otherwise, a fixed-effects model (Mantel-Haenszel method) [18] was applied. When considerable heterogeneity was identified, sensitivity analysis was performed by omitting one study in each turn to explore the influence of a single study on the overall pooled estimate. In some studies, the authors provided the Kaplan-Meier curves rather than HR and 95%CI; in these cases, we extracted data from the Kaplan-Meier curves using the method described by Tierney [19]. Publication bias was assessed using Begg's [20] and Egger's [21] test. A P-value of less than 0.05 was considered statistically significant. All analyses were performed using STATA version 12.0 (Stata Corporation, College Station, TX, USA).

## RESULTS

### Study identification and selection

**Figure 1:**
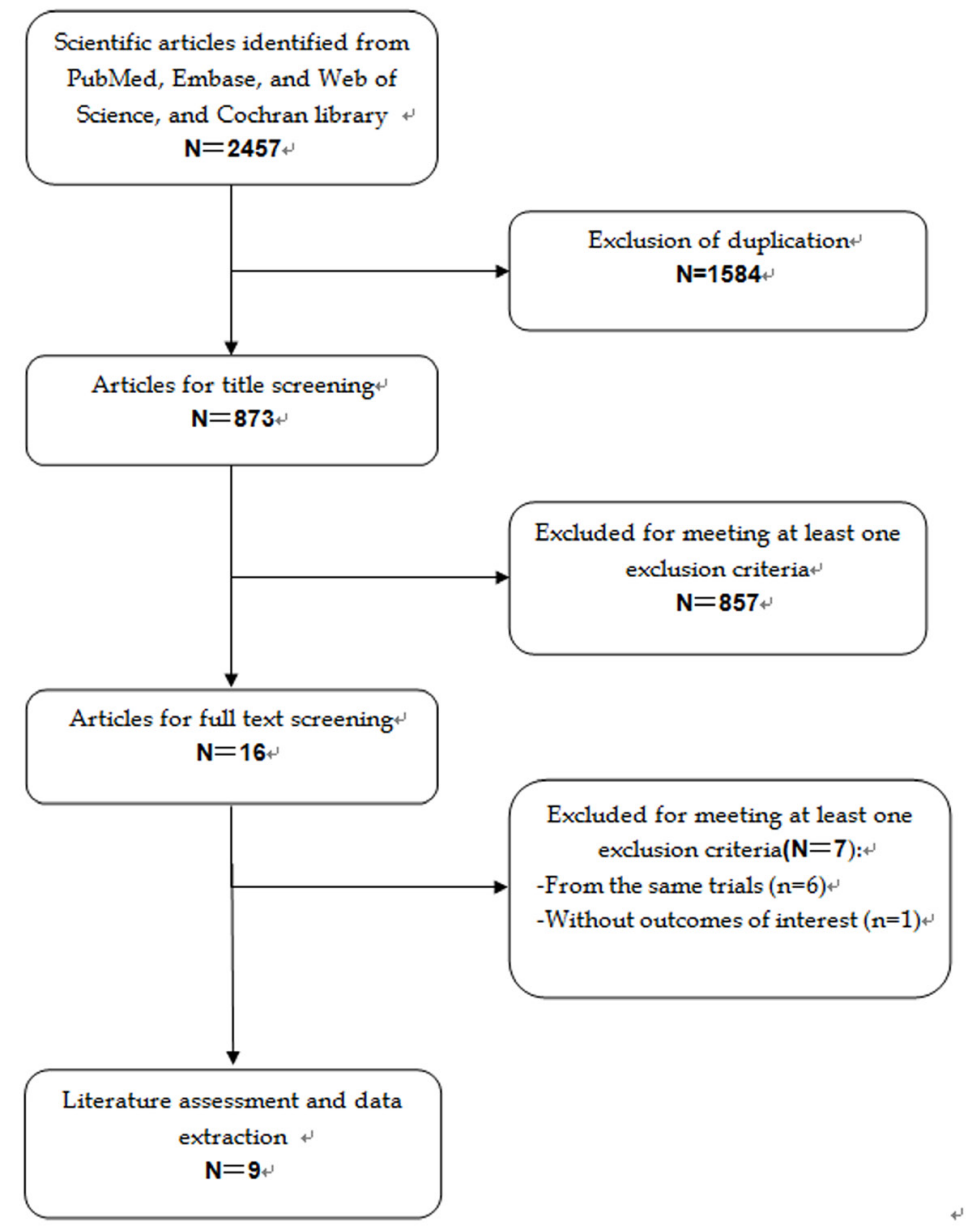
Eligibility of studies for inclusion in meta-analysis

A total of 2,457 potential articles were identified from the database search. Of these, 1,584 studies were excluded for duplicate records, and 857 studies were removed after a review of titles/abstracts. Then the full-text information of remaining 16 publications were scrutinized for further evaluation, and 7 of them were excluded because six were from three large-scale trials, and one did not provide outcomes of interest (Figure 1). Finally nine RCTs (involving 15,300 patients) [22–30] met the inclusion criteria, and they were included in this meta-analysis.

### Study characteristics

The main characteristics of the included studies were presented in Table 1. These trials were published between 2000 and 2016. The total number of included studies was 15,300 patients, ranging from 197 to 6,241 patients per study. The median follow up among these studies ranged from 13.3 to 120 months. Dosage and route of anastrozole in these included studies were consistent, in which anastrozole was orally administered with a dosage of 1 mg per day. However, for tamoxifen, all the studies reported a dosage of 20 mg per day, except the study conducted by Milla-Santos A, et al. [25], which reported a dosage of 40 mg per day.

**Table 1:** Baseline characteristics of patients in the trials included in the meta-analysis

Study	Treatment regimen	No. of patients	Age(mean ±SD, y)	Tumor size (cm)	ER status (positive/ negative/ unknown)	Median follow-up(m)
Margolese RG[22]	Anastrozole 1mg	1552	<60/≥60:731/821	<1.0/≥1.0/unknown: 528/389/635	NR	108(98.4-120)
	Tamoxifen 20mg	1552	<60/≥60:730/822	<1.0/≥1.0/unknown: 556/370/626	NR	108(98.4-120)
Aihara T[23]	Anastrozole 1mg	345	60(45-77)	<3/≥3: 274/71	321/24/0	98.4(2.4-134.4)
	Tamoxifen 20mg	351	60(44-82)	<3/≥3:278/73	326/25/0	98.4(2.4-134.4)
Bonneterre J[24]	Anastrozole 1mg	340	67(34-91)	NR	146/9/185	19
	Tamoxifen 20mg	328	66(41-92)	NR	142/2/184	19
Milla-Santos A[25]	Anastrozole 1mg	121	60.2(56-77)	NR	NR	13.3
	Tamoxifen 40mg	117	60.6(55-77)	NR	NR	13.3
Forbes J[26]	Anastrozole 1mg	1449	60.4(56.4-64.5)	1.3(0.7-2.2)	NR	86.4(67.2-106.8)
	Tamoxifen 20mg	1489	60.3(55.8-64.5)	1.3(0.7-2.2)	NR	86.4(67.2-106.8)
Masuda N[27]	Anastrozole 1mg	98	<60/≥60:98/0	NR	98/0/0	NR
	Tamoxifen 20mg	99	<60/≥60:99/0	NR	99/0/0	NR
Nabholtz JM[28]	Anastrozole 1mg	171	68(30-88)	NR	145/7/19	17.7
	Tamoxifen 20mg	182	67(40-92)	NR	156/5/21	17.7
Gnant M[29]	Anastrozole 1mg	451	45.5(27.6-56.5)	NR	421/16/14	47.8
	Tamoxifen 20mg	453	45(25.9-56.3)	NR	427/15/11	47.8
Cuzick J[30]	Anastrozole 1mg	3125	64.1±9.0	≤2/>2:1996/1103	NR	120(0-145)
	Tamoxifen 20mg	3116	64.1±9.0	≤2/>2:1959/1135	NR	120(0-145)

Abbreviations: NR, not reported; ER, oestrogen receptor

The ATAC trial was initially reported in 2002 by Baum M, et al. [31], updated in 2003 by Buzdar AU, et al. [32], and finally presented in 2010 by Cuzick J, et al. [30]. Thus, we included the latest version of study presented in 2010, and excluded the initial and updated versions. Similarly, for N-SAS BC03 trial, we included the latest version of study in 2014 conducted by Aihara T, et al. [23], and excluded the initial version in 2010 [33]. However, for TARGET trial, since the original study that reported in 2000 [24] presented the complete information, we included this version of study [24], and excluded the updated version in 2003 by Nabholtz JM, et al. [34].

### Risk of bias assessment and quality of evidence

**Figure 2:**
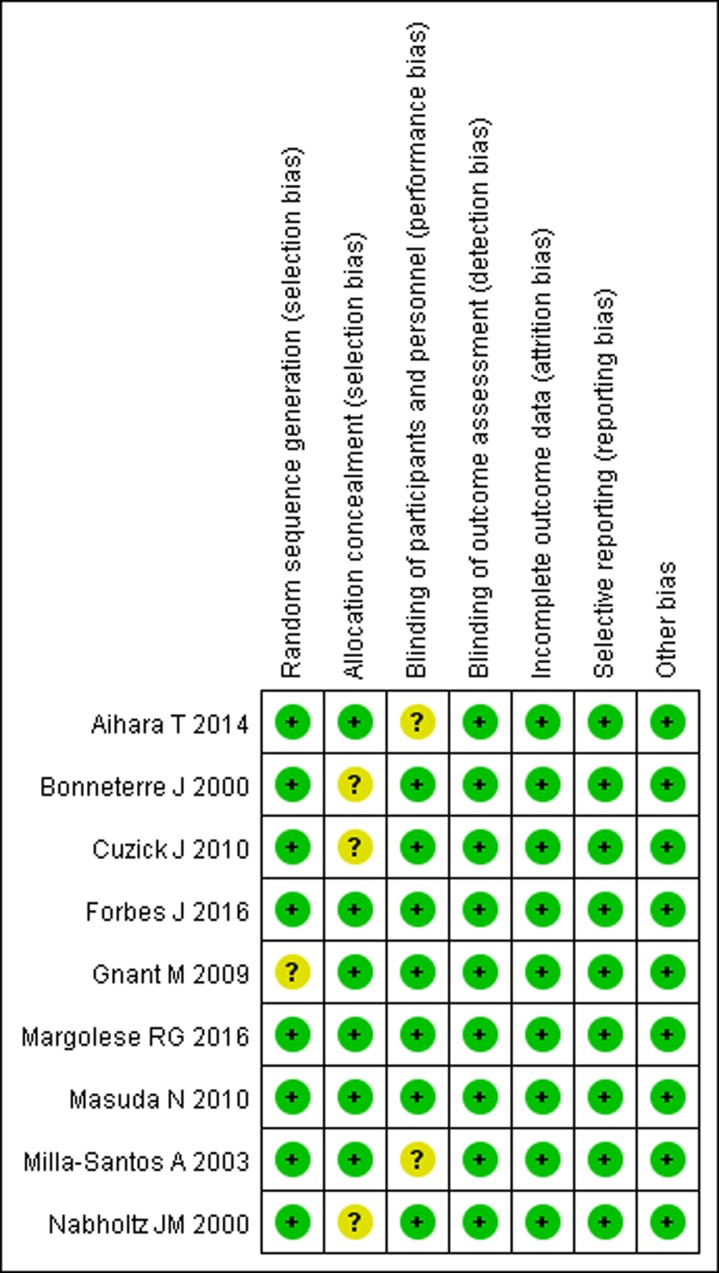
Risk of bias summary

The details of risk bias are summarized in Figure 2. Overall, three trials were considered as being at low risk of bias [22, 26, 27], and the remaining six as being unclear [23–25, 28–30]. Randomized sequence was adequately generated in eight trials [22–28, 30], and allocation sequence concealment was adequately reported in six studies [22, 23, 25–27, 29]. Blinding of participants and personnel and blinding of outcome assessors were reported in all these included trials.

The GRADE evidence profiles for these outcomes were shown in Table 2. The quality of evidence was high for DFS, RFS, OS, ORR and adverse events.

**Table 2:** GRADE evidence profile

Quality assessment							No of patients		Effect		Quality	Importance
													No of studies	Design	Risk of bias	Inconsistency	Indirectness	Imprecision	Other considerations	Anastrozole	Tamoxifen	Relative (95% CI)	Absolute
**Disease free survival (Better indicated by lower values)**												
8	randomized trials	no serious risk of bias	serious^1^	no serious indirectness	no serious imprecision	very strong association^2^	7483	7493	-	WMD 0.72 higher (0.55 to 0.94 higher)	⎕⎕⎕⎕ HIGH	CRITICAL
**Recurrence free survival (Better indicated by lower values)**												
7	randomized trials	no serious risk of bias	no serious inconsistency	no serious indirectness	no serious imprecision	strong association	8811	8865	-	WMD 0.86 higher (0.76 to 0.98 higher)	⎕⎕⎕⎕ HIGH	CRITICAL
**Over survival (Better indicated by lower values)**												
5	randomized trials	no serious risk of bias	no serious inconsistency	no serious indirectness	no serious imprecision	none	8079	8122	-	WMD 0.96 higher (0.77 to 1.21 higher)	⎕⎕⎕⎕ HIGH	IMPORTANT
**Overall response rate**												
5	randomized trials	no serious risk of bias	no serious inconsistency	no serious indirectness	no serious imprecision	none	267/730 (36.6%)	219/726 (30.2%)	RR 1.21 (1.05 to 1.39)	43 more per 1000 (from 3 more to 87 more)	⎕⎕⎕⎕ HIGH	IMPORTANT
**Adverse events**												
10	randomized trials	no serious risk of bias	no serious inconsistency	no serious indirectness	no serious imprecision	none	669/4660 (14.7%)	821/4651 (17.7%)	RR 0.77 (0.47 to 1.25)	41 fewer per 1000 (from 94 fewer to 44 more)	⊕⊕⊕⊕HIGH	IMPORTANT

1Substantial heterogeneity (I^2^ = 92.3%) was found

2A total of 14,976 patients were enrolled.

### Disease free survival

**Figure 3:**
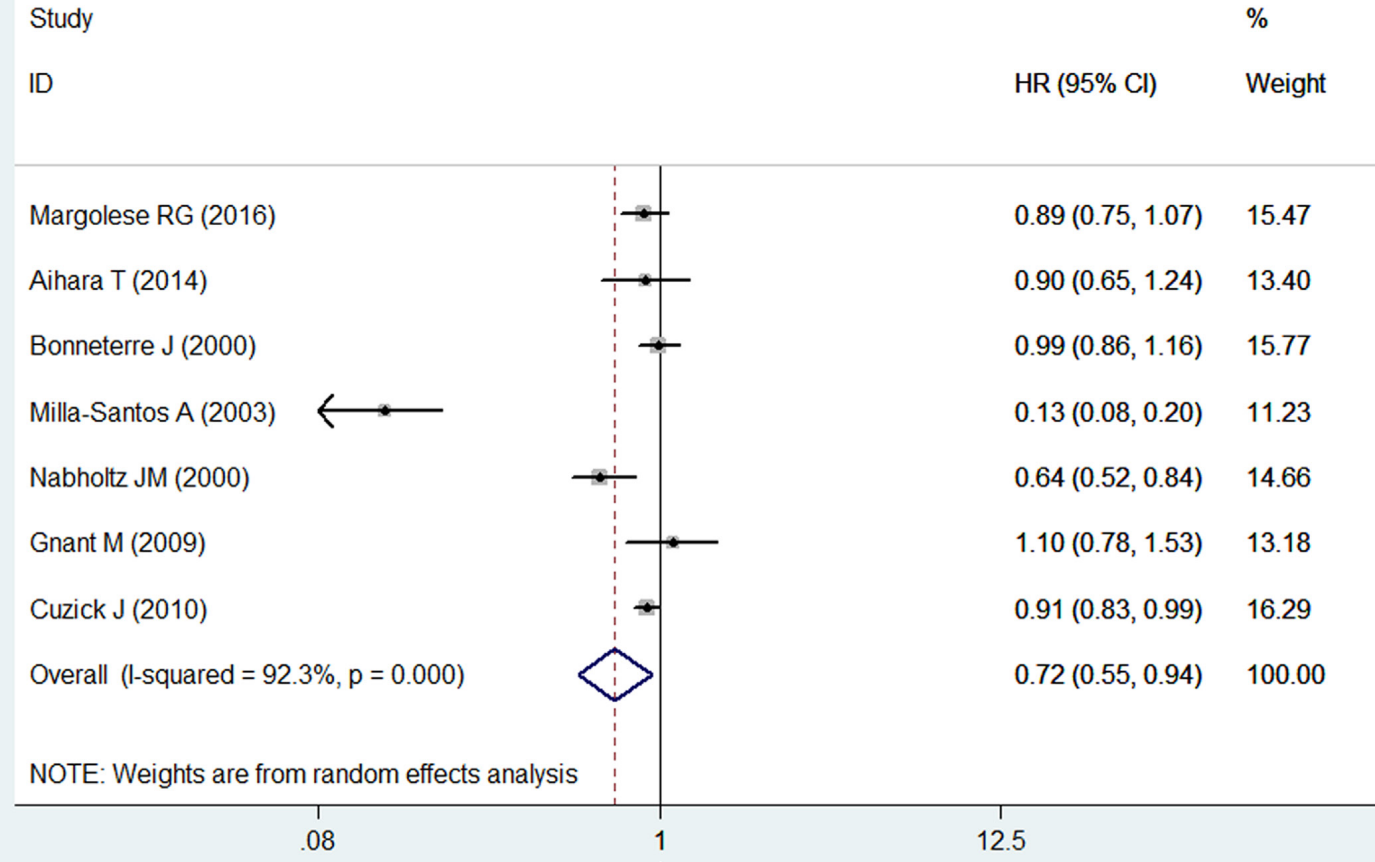
Forest plot showing the effect of anastrozole *versus* tamoxifen on disease free survival

Seven studies reported the data for DFS [22–25, 28–30]. The pooled estimates demonstrated that anastrozole significantly prolonged DFS compared with tamoxifen (HR = 0.72, 95%CI: 0.55-0.94; *P* = 0.016) (Figure 3). The test for heterogeneity was significant (*P* < 0.001, *I*2 = 92.3%). Therefore, we conducted sensitivity analysis to explore the potential heterogeneity. A trial conducted by Milla-Santos A, et al. [25], reported patients with postmenopausal, hormone-dependent, advance breast cancer and they were treated with anastrozole 1mg or tamoxifen 40 mg per day. When we excluded this trial, the overall estimates did not change substantially (HR = 0.89, 95%CI: 0.80-1.00; *P* = 0.050), but no evidence of heterogeneity was found (*P* = 0.077, *I*2 = 47.4%). The Begg and Egger's test showed that there was no potential publication bias among the included studies (Egger's test, *P* = 0.213; Begg's test, *P* = 0.133).

### Recurrence free survival

**Figure 4:**
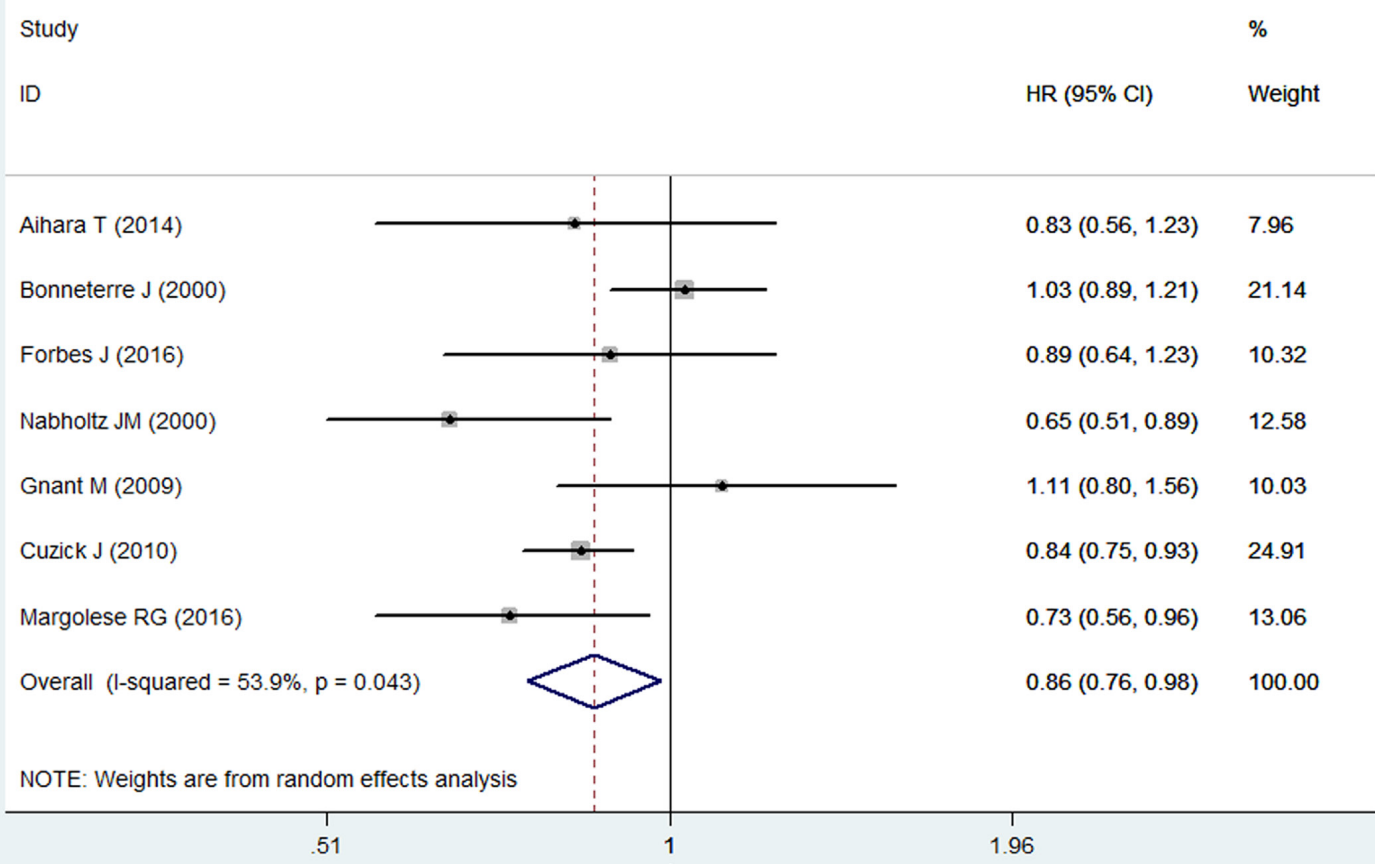
Forest plot showing the effect of anastrozole *versus* tamoxifen on recurrence free survival

Seven studies reported the data for RFS [22–24, 26, 28–30]. The aggregated results of these studies indicated that, anastrozole was associated with a significantly improved RFS than tamoxifen (HR = 0.86, 95%CI: 0.76-0.98; *P* = 0.024) (Figure 4). There was a little statistical heterogeneity among the included studies (*P* = 0.043, *I*2 = 53.9%). The Begg and Egger's test revealed no existence of publication bias among the included studies (Egger's test, *P* = 0.806; Begg's test, *P* = 0.881).

### Over survival

**Figure 5:**
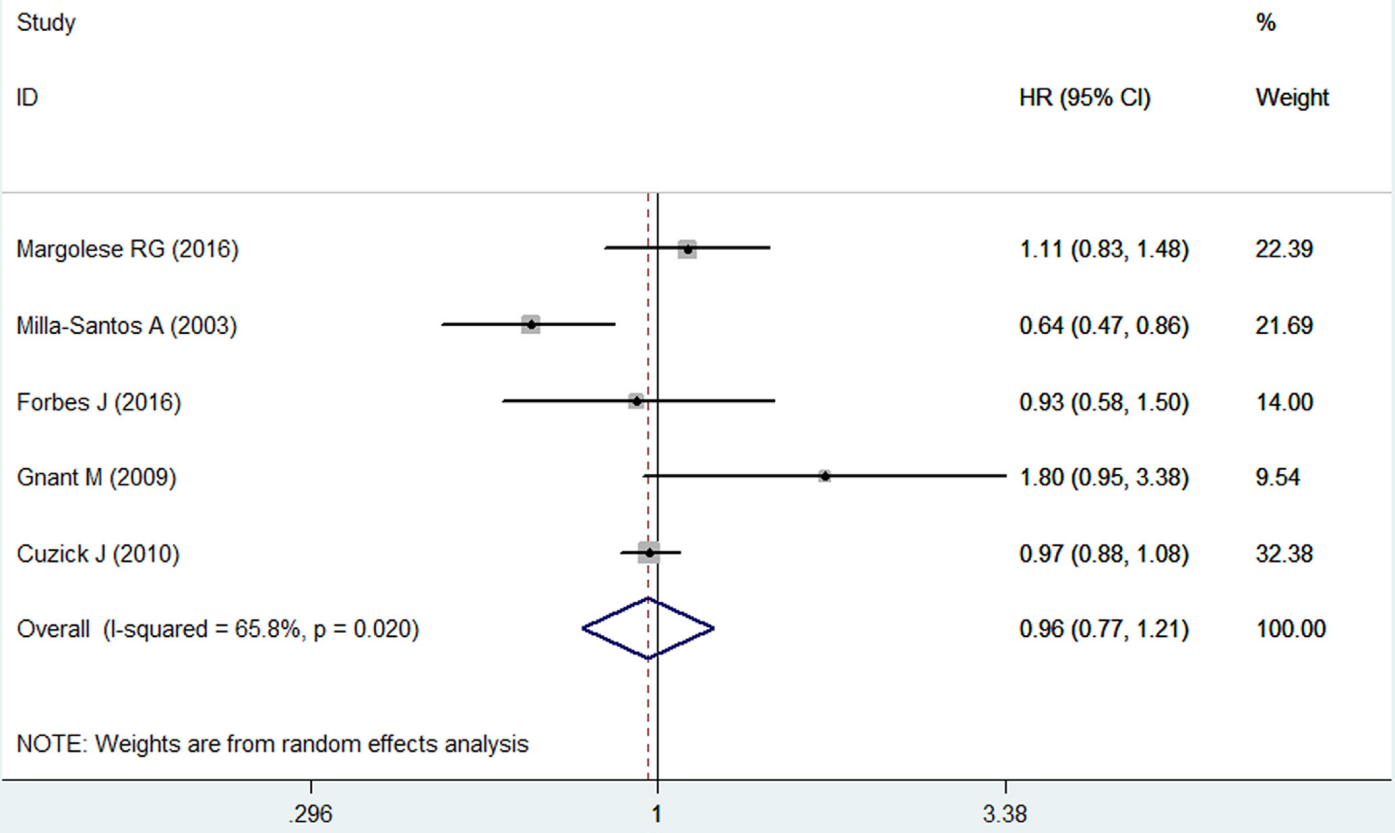
Forest plot showing the effect of anastrozole *versus* tamoxifen on overall survival

Eight of the nine included studies reported OS outcome, but only five provided available data for analysis [22, 25, 26, 29, 30]. The pooled results showed that anastrozole did not significantly improve OS as compared with tamoxifen (HR = 0.96, 95%CI: 0.77-1.21; *P* = 0.751) (Figure 5). The test for heterogeneity was significant (*P* = 0.020, *I*2 = 65.8%). Therefore, we conducted the sensitivity analysis. When we removed trial conducted by Milla-Santos A, et al. [25], the pooled results changed slightly (HR = 1.00, 95%CI: 0.91-1.09; *P* = 0.924), but no evidence of heterogeneity was observed among the remaining studies (*P* = 0.239, *I*2 = 28.9%).

### Overall response rate

**Figure 6:**
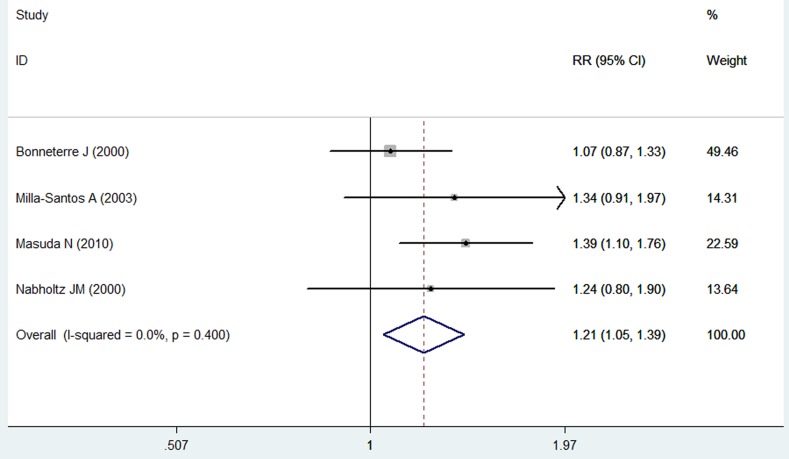
Forest plot showing the effect of anastrozole *versus* tamoxifen on overall response rate

Four studies reported the data for ORR [24, 25, 27, 28]. The pooled results suggested that patients with breast cancer who were treated with anastrozole had a higher ORR than those treated with tamoxifen (RR = 1.21, 95% CI: 1.05-1.39; *P* = 0.009) (Figure 6). The test for heterogeneity was not significant (*P* = 0.400, *I*2 = 0.0%).

### Adverse events

All the studies reported the data for adverse events [22–30]. The incidences of adverse events in the anastrozole and tamoxifen groups were 14.4% and 17.7%, respectively. Pooled estimates showed that, anastrozole had a comparable incidence of adverse events as tamoxifen (RR = 0.77, 95%CI: 0.47-1.25; *P* = 0.303). The most common adverse events are listed in Table 3. The pooled results demonstrated that, compared with tamoxifen, anastrozole was associated with a significantly higher incidence of arthralgia (RR = 1.55, 95%CI: 1.20-1.99; *P* = 0.001), bone pain (RR = 1.31, 95%CI: 1.05-1.62; *P* = 0.015), but a lower incidence of vaginal bleeding (RR = 0.51, 95%CI: 0.28-0.93; *P* = 0.029), vaginal discharge (RR = 0.31, 95%CI: 0.12-0.82; *P* = 0.017), and thromboembolic events (RR = 0.39, 95%CI: 0.28-0.55; *P* < 0.001).

**Table 3:** Summary of the risk ration (RR) of adverse events

Adverse events	Risk ratio (RR)	95% CI	*P* value
Arthralgia	1.55	1.20-1.99	0.001
Bone pain	1.31	1.05-1.62	0.015
Vaginal bleeding	0.51	0.28-0.93	0.029
Vaginal discharge	0.31	0.12-0.82	0.017
Thromboembolism	0.39	0.28-0.55	<0.001
Nausea	1.00	0.82-1.23	0.987
Hot flush	0.95	0.82-1.11	0.551
Hypertension	0.92	0.53-1.59	0.756
Bone fracture	1.16	0.99-1.35	0.072
Constipation	0.62	0.38-1.02	0.059
Diarrhea	1.35	0.81-2.24	0.245

## DISCUSSION

This study is a meta-analysis with the objective of comparing the efficacy and safety of anastrozole *versus* tamoxifen as adjuvant treatment for breast cancer. Our study suggested that, anastrozole was associated with a significantly improvement in DFS, RFS and ORR than tamoxifen, but was not in OS. Moreover, anastrozole induced a higher incidence of arthralgia and bone pain, as well as a lower incidence of vaginal bleeding, vaginal discharge, and thromboembolic events than tamoxifen. These results suggested that women with breast cancer could benefit from treatment of anastrozole.

There has been one published meta-analysis that assessed the effect of switching to anastrozole after 2-3 years of tamoxifen treatment, compared with continuing on tamoxifen for 5 years [35]. In that study, the authors included three clinical trials: the ABCSG-8 trial, Arimidex-Nolvadex (ARNO 95), and the Italian Tamoxifen Anastrozole (ITA) [35]. All the postmenopausal women enrolled had histologically confirmed, hormone-sensitivity early stage breast cancer, and were randomized to receive 1 mg/day anastrozole after 2-3 years of tamoxifen treatment or to continued 20 or 30 mg/day tamoxifen [35]. Their results suggested that, compared with continuing on tamoxifen, switching to anastrozole was associated with a significant improvements in DFS (HR = 0.59, 95%CI: 0.48-0.74; *P* < 0.001), event-free survival (HR = 0.55, 95%CI: 0.42-0.71; *P* < 0.001), and distant RFS (HR = 0.61, 95%CI: 0.45-0.83; *P* = 0.002) [35]. These results were comparable to data from our meta-analysis. However, with regard to the OS, we observed a converse result with this previously published meta-analysis. In the present study, the anastrozole treatment had a similar OS with tamoxifen. While, in the previous meta-analysis, the treatment of switching to anastrozole resulted in an improved OS than continuing on tamoxifen (HR = 0.71, 95%CI: 0.52-0.98; *P* = 0.004) [35].

In this meta-analysis, anastrozole significantly reduced the risk of disease progression by 28% (HR = 0.72, 95%CI: 0.55-0.94; *P* = 0.016). This finding was consistent with the result of the ATAC trial [30], which also reported a beneficial effect of anastrozole over tamoxifen. ATAC trial was a randomized, multicenter trial that compared the efficacy and safety of anastrozole (1mg) with tamoxifen (20mg), both given orally every day for 5 years, as adjuvant treatment for women with early-stage breast cancer [30]. After a median follow up of 120 months (range 0-145), 30.5% (953/3125) and 32.8% (1022/3116) of patients in the anastrozole and tamoxifen groups developed disease progression, respectively. The corresponding HR for DFS was 0.91(95%CI: 0.83-0.99; *P* = 0.04) [30]. Moreover, this survival benefit was also observed in patients with hormone-receptor positive disease, which reported a 14% less of risk for developing disease progression (HR = 0.86, 95%CI: 0.78-0.95; *P* = 0.03) [30].

However, the improvement of DFS associated with anastrozole was not observed in a phase 3 National Surgical Adjuvant Breast and Bowel Project (NSABP) B-35 trial [22]. In that trial, postmenopausal women with hormone-positive ductal carcinoma *in situ* were randomly assigned to receive either oral anastrozole 1 mg per day or tamoxifen 20 mg per day [22]. At the end of 10-years’ follow up, the DFS rate in these two groups were 82.7% and 77.9%, respectively [22]. Although there were strong trends toward improved DFS in the anastrozole group, this difference was not statistically significant (HR = 0.89, 95%CI: 0.75-1.07; *P* = 0.21). Furthermore, when the DFS outcome was analyzed based on age, significant effect was observed in women aged younger than 60 years (HR = 0.69, 95%CI: 0.51-0.93; *P* = 0.02) [22]. Thus, the authors concluded that young women would achieve a better DFS than older women, although there was no obvious biological explanation for this difference [22]. Owning to the limited data, we did not perform subgroup analysis to explore whether the effect of anastrozole was only restricted to women who were younger than 60 years old.

Regarding the RFS, our study showed that patients who were treated with anastrozole had an improved RFS compared with tamoxifen (HR = 0.86, 95%CI: 0.76-0.98; *P* = 0.024). This result was in line with the data from the ATAC trial [30]. In that study, the RFS rate in the anastrozole and tamoxifen group was 19.6% and 23.0%, respectively (HR = 0.84, 95CI: 0.75-0.93). However, this survival benefit was only observed in subpopulation patients who had hormone receptor positive disease [30]. Among these patients, the recurrence rates in these two groups were 17.1% and 21.5%, respectively, and the HR for RFS was 0.79 (95%CIs: 0.70-0.89).

Contrast to the ATAC trial, IBIS-II DCIS trial did not find a statistically significant difference in RFS [26]. IBIS-II DCIS trial was a double-blind, multicenter, randomized placebo-control trial, which enrolled postmenopausal women with hormone-receptor-positive DCIS [26]. In that study, 67 (4.62%) patients in the anastrozole group and 77 (5.17%) patients in the tamoxifen group developed breast cancer recurrences, respectively [26]. The corresponding value for HR with 95%CIs was 0.89 (0.64-1.23) [26]. Furthermore, when the authors used the univariate analyses to calculate HR values, the comparable effect was still present between the two groups [26]. This might be explained by the history of DCIS in these patients. According to the previous studies, DCIS had been recognized as a precursor of invasive cancer, and patients with DCIS was more likely to develop invasive breast cancer [36, 37]. Another interesting finding in the IBIS-II DCIS trial was that, the improved RFS was only observed in ER-positive patients, but not in ER-negative patients. Among the 86 ER-positive recurrences, 30 (2%) occurred in the anastrozole group compared with 56(4%) in the tamoxifen group (HR = 0.55, 95%CI: 0.35-0.86), which indicated a benefit in favor of anastrozole [26]. Whereas, for the 30 ER-negative recurrences, 17 (1%) were in the anastrozole group compared with 13 (< 1%) in tamoxifen group (HR = 1.34, 95CI: 0.65-2.75), which suggested a similar effect between the two treatments [26]. Previous studies have suggested that the hormone receptor status was an important factor in predicting the treatment effects of endocrine therapy: patients with hormone receptor positive tumors would achieve the greatest effect, whereas those with hormone receptor negative tumors were questionable [38, 39]. This could explain why the beneficial effect of anastrozole was only observed in ER-positive patients but not in ER-negative patients.

Among the included studies, the improvement of OS was only observed in a phase III trial [25], in which anastrozole was used as first-line therapy. In this trial, 121 and 117 patients were randomized to receive anastrozole or tamoxifen, respectively. At the time of data cutoff, 60% of patients died in the anastrozole group as compared with 89% in the tamoxifen group [25]. The median OS times for the two groups were 17.4 and 16.0 months, respectively (HR = 0.64, 95CI: 0.47-0.86; *P* = 0.003) [25], indicating that anastrozole significantly reduced the risk of death by 36% as compared with tamoxifen. However, in the Austrian Breast and Colorectal Cancer Study Group trial 12 (ABCSG-12) [29], anastrozole resulted in an opposite result. In that study, anastrozole had similar effects with tamoxifen in OS, and the HR for OS was 1.80 (95%CI: 0.95-3.38; *P* = 0.007) [29]. Although there was a strong trend for improved OS in tamoxifen group than anastrozole group, the difference between them was not significant [29]. The precise reason for the prolonged OS in favor of tamoxifen is not clear, however, it was speculated that the absence of palliative treatment with aromatase inhibitors in the anastrozole group could affect the OS [29, 40].

Additionally, the BMI may also have potential impact on the OS. In a retrospective analysis of ABCSG-12 trial [41], the authors found a greater OS in favor of tamoxifen plus gosereline over anastrozole plus goserelin. However, this beneficial effect was only observed in the subset of patients with BMI > 25 kg/m^2^, but not in those with BMI < 25 kg/m^2^ [41]. Similarly, in the retrospective analysis from the ATAC trial [42], obese women (BMI > 30 kg/m^2^)treated with anastrozole had a poorer overall prognosis than those with BMI lower than 23 kg/m^2^ [42]. It seemed that patients with BMI > 25 kg/m^2^ would achieve a better OS when they were treated with tamoxifen, while those with BMI < 25 kg/m^2^ might obtain a greater OS when they were treated with anastrozole. This also could explain the better efficacy for tamoxifen than for anastrozole in the ABCSG-12 trial [41], since 33.0% of patients in that study had a BMI higher than 25 kg/m^2^.

In this meta-analysis, we found that the overall incidence of adverse events was similar between the anastrozole and tamoxifen groups. Patients treated with anastrozole had a significantly fewer vaginal bleeding, vaginal discharge, and thromboembolic events than did those treated with tamoxifen. Most of these drug-related adverse events were mainly mild to moderate in severity. In the overall analysis, anastrozole seems to have a preferable safety profile to tamoxifen for adverse events.

This meta-analysis has several potential limitations that should be considered. First, there was substantial heterogeneity in DFS outcomes among the included studies. However, it should not be surprising when considering the differences in region, ethnicity, BMI, hormone receptor status, tumor grade, the dosage of tamoxifen, and duration of follow up. These factors may result in an overestimation or underestimation of the true DFS. Second, due to the sparse reporting among these trials, we did not compare the effects of anastrozole over tamoxifen in subset of patients with different ER status, hormone receptor status, and BMI. Third, because all of these trials were partly funded by the pharmaceutical industry, we could not exclude the possibility that their results were affected by the inherent conflict of interest and possible bias. Therefore, cautions should be taken when interpreting our results.

In conclusion, our meta-analysis indicated that, anastrozole was associated with an improvement in DFS, RFS and ORR in the treatment of patients with breast cancer, but not in OS. Moreover, anastrozole also produced a comparable incidence of adverse events with tamoxifen. However, given the potential limitations in this study, additional large-scale and well-designed RCTs are needed to substantiate our findings, and investigate the effects of anastrozole in subpopulation patients with different ER status, hormone receptor status, and BMI.
